# Antifungal effects and potential mechanism of bortezomib in combination with fluconazole against *Candida albicans*

**DOI:** 10.3389/fcimb.2026.1856948

**Published:** 2026-09-17

**Authors:** Yuting Wang, Bing Wang, Jinfeng Li, Shuqing Ma, Peng Liu, Zhitao Teng, Haijie Zou, Rinan Cong, Xiaojing Sui, Xiaohui Li, Yingjie Cui

**Affiliations:** 1 Department of Pharmacy, Weihai Municipal Hospital, Cheeloo College of Medicine, Shandong University, Weihai, Shandong, China; 2 Clinical Laboratory Center, Weihai Municipal Hospital, Cheeloo College of Medicine, Shandong University, Weihai, Shandong, China; 3 Department of Cardiovascular Medicine, Weihai Municipal Hospital, Cheeloo College of Medicine, Shandong University, Weihai, Shandong, China

**Keywords:** antifungal effect, antifungal mechanisms, bortezomib, *Candida albicans*, combination therapy, fluconazole

## Abstract

The clinical management of *Candida albicans* (*C. albicans*) infections presents considerable problems, intensified by the few therapeutic choices available for this disease. The increasing resistance to fluconazole (FLC) following its widespread clinical use underscores the need for novel therapeutic strategies. Drug repurposing represents a promising approach to expand the antifungal armamentarium, and bortezomib (BTZ), a proteasome inhibitor originally approved for multiple myeloma, has emerged as a candidate for this purpose. This work systematically assessed the synergistic effects of fluconazole (FLC) with bortezomib (BTZ) on the susceptibility of *C. albicans*, focusing specifically on the planktonic and biofilm development phases. The results exhibited a synergistic antifungal effect of BTZ and FLC against FLC-resistant *C. albicans* in planktonic cells. The minimal inhibitory concentration (MIC) of BTZ decreased from 64-128 µg/ml to 8 µg/ml, whereas the MIC of FLC reduced from >128 µg/ml to 0.25 µg/ml, resulting in a fractional inhibitory concentration index (FICI) of less than 0.5. For FLC-susceptible *C. albicans* strains, the medication combination exhibited an indifferent interaction, with FICI above 0.5. The combination exhibited synergistic effects against *C. albicans* during the early stages of biofilm formation (4 and 8 h). The sessile minimum inhibitory concentration (sMIC) of FLC significantly lowered from ≥128 µg/mL to 0.25 µg/mL, whereas the sMIC of BTZ diminished from ≥128 µg/mL to 8-16 µg/mL, with a FICI of less than 0.5, signifying significant synergy. Time-kill assays revealed a delayed but sustained inhibitory effect against planktonic cells (24–48 h), while CFU enumeration confirmed a concentration-dependent reduction in biofilm viability, with the higher concentration (0.25 + 16) maintaining efficacy through 24 h. *In vivo*, using the *Galleria mellonella* (*G. mellonella*) infection model, the FLC+BTZ combination significantly improved larval survival, reduced fungal burden, and alleviated tissue damage compared with FLC monotherapy. Mechanistic studies demonstrated that this synergistic effect was mediated by multiple mechanisms, including the suppression of hyphal growth, inhibition of efflux pump activity, and intracellular formation of reactive oxygen species (ROS). These findings highlight the potential of drug repurposing in combating antifungal resistance and provide mechanistic insights into overcoming *C. albicans* drug resistance.

## 1 Introduction

Over the past several years, the incidence of *C. albicans* infections has exhibited a consistent upward trend (Parambath et al., 2024). *C. albicans* is the main opportunistic pathogenic fungus in humans (Macias-Paz et al., 2023). Its pathogenicity is largely attributed to its remarkable morphogenic plasticity, the ability to switch among yeast, hyphal, and pseudohyphal forms in response to different host environments (Sharma et al., 2024). This concerning epidemiological shift is primarily attributable to the increasing global prevalence of immunocompromised individuals, the widespread and often inappropriate use of antibiotics and immunosuppressive agents, and the expanding application of radiotherapy and chemotherapy in the management of malignant neoplasms (Pfaller and Diekema, 2007). The escalating public health burden of pathogenic fungal infections and the current treatment gaps in *C. albicans* infections further highlight the urgent need for novel therapeutic strategies (Dong et al., 2026; Miao et al., 2026). Azole antifungals, especially fluconazole (FLC), have become essential in the treatment of *C. albicans* infections owing to their advantageous pharmacological characteristics, which encompass high efficacy, low toxicity, superior absorption, and cost-effectiveness. Nonetheless, azole-resistant strains have arisen due to the increasing clinical application of FLC, and the mechanisms underlying such resistance have been extensively studied (Kaur and Nobile, 2023; Sharma et al., 2024),necessitating the development of novel therapeutic strategies to tackle this escalating issue (Krcmery and Barnes, 2002; Whaley et al., 2017). A noteworthy advancement in the field is the emergence of combination therapy, which presents a viable way to improve antifungal effectiveness, circumvent resistance, and minimize potential toxicity.

Drug repurposing offers a promising strategy to expand the antifungal arsenal by identifying new indications for existing drugs. Bortezomib (BTZ), a first-generation proteasome inhibitor, received FDA approval in 2003 for the treatment of multiple myeloma and has been thoroughly investigated in conjunction with melphalan and prednisone for this purpose (Palumbo et al., 2016; Attal et al., 2017; Tan et al., 2018; Mateos et al., 2020; Yamamoto and Egashira, 2021). Originally developed as an antineoplastic agent, BTZ has recently drawn attention as a candidate for drug repurposing due to its reported antimicrobial potential. A previous study reported that BTZ enhances FLC’s antifungal activity, but only tested a standard strain (*C. albicans SC5314*). However, the combined effects on clinically isolated FLC-sensitive/resistant *C. albicans* strains, biofilm activity, and underlying antifungal mechanisms, has not been reported (Kaneko et al., 2013). Emerging evidence suggests that antifungal combination or alternation strategies can effectively reduce drug-tolerant populations, further supporting the rationale for exploring novel synergistic approaches (Cai et al., 2026).To address these gaps, we extended the study to evaluate the synergistic effects of BTZ and FLC on a wider range of clinical isolates, including both sensitive and resistant strains, as well as their activity against *C. albicans* biofilms. In addition, we explored the potential synergistic effects of BTZ and FLC against *C. albicans* by targeting multiple antifungal mechanisms, including efflux pump inhibition, intracellular reactive oxygen species (ROS) accumulation and suppression of the yeast-to-hyphal transition.

## 2 Materials and methods

### 2.1 Isolates, growth media, drugs

This investigation included six strains susceptible to FLC and six strains resistant to FLC, as detailed in **Table 1**. Professor Changzhong Wang from the School of Integrated Traditional and Western Medicine at Anhui University of Traditional Chinese Medicine in Hefei, China, gave the following four strains: CA103, CA137, CA632, and CA20003. The residual strains were acquired from the clinical laboratory of the First Hospital Affiliated with Shandong First Medical University in Jinan, China. The quality control strain *C. albicans* ATCC 10231 was supplied by the Institute of Pharmacology, School of Pharmacy, Shandong University. All strains were preserved at -80 °C and grown a minimum of three times on Sabouraud Dextrose Agar (SDA) solid medium.

**Table 1:** Antifungal activities of bortezomib (BTZ) used as monotherapy or in combination with fluconazole (FLC) against *Candida albicans*.

Strains^a^	MIC_80_ (μg/mL)^b^				FICI^b^	Interaction^c^
	Alone		Combined			
	FLC	BTZ	FLC	BTZ		
CA10(R)	>128	64	0.25	8	0.127	SYN
CA16(R)	>128	64	0.25	8	0.127	SYN
CA103(R)	>128	128	0.25	8	0.064	SYN
CA137(R)	>128	128	0.25	8	0.064	SYN
CA632(R)	>128	128	0.25	8	0.064	SYN
CA20003(R)	>128	128	0.25	8	0.064	SYN
CA4(S)	0.5	128	0.25	4	0.531	NI
CA8(S)	0.5	>128	0.25	4	0.531	NI
CA14(S)	0.25	128	0.25	2	1.016	NI
CA19(S)	0.25	128	0.25	2	1.016	NI
CA20(S)	0.25	128	0.25	2	1.016	NI
CA129(S)	0.25	128	0.25	2	1.016	NI

^a^ CA, Candida albicans.

^b^ FICI, fractional inhibitory concentration index.

MIC denotes minimum inhibitory concentration of drug that inhibited fungal growth by 80% compared with the growth control.

MIC values and FICIs are shown as the median of three independent experiments.

^c^ SYN, synergism; NI, no interaction.

BTZ was acquired from *Beijing Psaitong Biotech Co., Ltd. (Beijing, China)*, and FLC was obtained from *Dalian Meilun Biotech Co., Ltd. (Dalian, China)*. The BTZ (2560 µg/mL) solution needs to be created by dissolving it in dimethyl sulfoxide (DMSO), whereas the FLC (2560 µg/mL) solution should be prepared by dissolving it in distilled water, following the manufacturer’s instructions. Stock solutions must be maintained at -20 °C until used. The Ethics Committee of Weihai Municipal Hospital accepted this study (No. 2022091).

### 2.2 *In vitro* antifungal efficacy of BTZ and FLC against *C. albicans*

#### 2.2.1 Assessment of minimum inhibitory concentrations of planktonic cells

The antifungal efficacy of FLC and BTZ against *C. albicans* was assessed via the microdilution technique in 96-well flat-bottom microliter plates, in line with the Clinical and Laboratory Standards Institute standard M27-A3 (CLSI, M27-A3). The ultimate concentrations of FLC and BTZ varied from 0.25 to 128 µg/mL and 2 to 128 µg/mL, respectively. Each well contained 100 µL of a yeast cell suspension (2×10³ CFU/mL), to which 50 µL of the corresponding antifungal drugs were added, yielding a total volume of 200 µL per well. A drug-free well containing solely RPMI 1640 medium functioned as the control group. The plates were incubated at 35 °C for 24 hours, and the minimum inhibitory concentration (MIC) was assessed using visual examination and optical density measurement at 600 nm utilizing a microplate reader according to the CLSI M27-A3 guideline. The MIC was established as the minimum medication concentration that suppressed growth by 80% relative to the control group (Khan and Ahmad, 2011; Su et al., 2020). The fractional inhibitory concentration index (FICI) was employed to evaluate the interactions of medication combinations, calculated as follows: FICI = FIC_FLC_ + FIC_BTZ_ = (MIC_80_ of FLC in combination/MIC_80_ of FLC alone) + (MIC_80_ of BTZ in combination/MIC_80_ of BTZ alone). The FICI was elucidated as follows: FICI ≤ 0.5 signifies synergy, FICI > 4.0 denotes antagonism, and 0.5 < FICI ≤ 4.0 reflects the absence of interaction. To guarantee reproducibility, the experiment was conducted on three separate occasions (Lewis, 2002; Odds, 2003).

#### 2.2.2 Assessment of sMICs for *C. albicans* biofilms

To test the sMIC values of *C. albicans* biofilms, four strains were randomly chosen from the previously analyzed 12 strains of *C. albicans*, including two FLC-sensitive strains and two FLC-resistant strains. Four representative strains, including two FLC-susceptible strains (CA4 and CA8) and two FLC-resistant strains (CA10 and CA16), were selected to cover both susceptible and resistant phenotypes. The sMIC values of FLC in conjunction with BTZ against the biofilms of the selected strains (CA4, CA8, CA10, and CA16) were assessed using 96-well microtiter plates. The studies were conducted at different time points, with modifications to the previously described methods as necessary (Ramage et al., 2001). A cell suspension was produced at a concentration of 1 × 10³ CFU/mL, and 200 µL was dispensed into each well of a 96-well culture plate. Plates were incubated at 35 °C for 4, 8, 12, and 24 hours to allow initial adhesion and subsequent biofilm maturation. The wells were subsequently rinsed three times with 200 µL of sterile phosphate-buffered saline (PBS) to eliminate planktonic and non-adherent cells. Subsequent to this, diverse concentrations of the medications were introduced into the wells, and the plates were incubated at 35 °C for an additional 24 hours. The final concentrations of FLC varied from 0.25 to 128 µg/mL, whereas those of BTZ ranged from 2 to 128 µg/mL. The untreated well was designated as the growth control. The sMIC_80_ values were ascertained by a metabolic assay employing 2,3-bis(2-methoxy-4-nitro-5-sulfophenyl)-2H-tetrazolium-5-carboxanilide (XTT) reduction (Zhong et al., 2017). sMIC_80_ was determined as the minimal concentration of the evaluated drug that achieved an 80% decrease in XTT absorption relative to the growth control group. The interaction between BTZ and FLC against *C. albicans* biofilms was assessed utilizing the previously stated FICI model. This experiment was performed independently on three occasions.

#### 2.2.3 Biofilm CFU enumeration assay

Biofilms of *C. albicans* CA10 (1 × 10^6^ CFU/mL) were formed in 96-well plates in RPMI 1640 at 37 °C for 90 min to allow initial adhesion. After removing non-adherent cells by washing with PBS, biofilms were treated with FLC (0.25 μg/mL), BTZ (8 or 16 μg/mL), or their combinations (0.25 + 8 and 0.25 + 16) for 4, 8, 12, and 24 h. At each time point, biofilms were washed with PBS, scraped, and sonicated in PBS to disperse cell clumps. Cell suspensions were serially diluted and plated on Sabouraud dextrose agar (SDA) plates. CFU per well was calculated after 24–48 h of incubation. The limit of detection (LOD) was defined as one colony at the lowest dilution plated, and samples with no colonies were assigned LOD/2 for log_10_ transformation. Data were log_10_-transformed and expressed as mean ± SD from three independent experiments. Statistical significance was determined by two-way repeated-measures ANOVA with Dunnett’s *post hoc* test using GraphPad Prism 8.3.0 to compare the combination groups with FLC alone. A p-value of < 0.05 was considered statistically significant.

#### 2.2.4 Time-kill assay

Time-kill curves were performed with an initial inoculum of approximately 1 × 10^5^ CFU/mL of *C. albicans* CA10 in RPMI 1640 at 35°C with shaking (Gong et al., 2019). The culture was treated with FLC (0.25 μg/mL), BTZ (8 μg/mL), or their combinations (0.25 + 8). At 0, 6, 12, 24, and 48 h, aliquots were removed, serially diluted, and plated on SDA. CFU/mL was counted after 24–48 h of incubation. Samples with no colonies were assigned LOD/2 for log_10_ transformation. Three independent experiments were performed. Statistical significance was determined by two-way repeated-measures ANOVA with Dunnett’s *post hoc* test using GraphPad Prism 8.3.0.

### 2.3 Antifungal Effects of BTZ and FLC Against *C. albicans in vivo*

#### 2.3.1 Galleria mellonella (*G. mellonella*) survival assay

The *in vivo* antifungal efficacy of the FLC+BTZ combination was evaluated using the *G. mellonella* infection model. Healthy larvae (220–280mg) with uniform size and no melanization were randomly divided into four groups (n = 20 per group): control (PBS), FLC alone, BTZ alone, and FLC+BTZ combination. A suspension of *C. albicans* CA10 (5 × 10^8^ CFU/mL) was prepared in sterile PBS, and 10 μL was injected into the last left proleg of each larva. Two hours post-infection, 10 μL of drug solutions were injected into the last right proleg at the following concentrations: FLC (160 μg/mL), BTZ (4 μg/mL), and FLC+BTZ (160 + 4 μg/mL). The concentrations of FLC and BTZ were selected based on our previous studies and the clinically approved human dose of bortezomib (1.3 mg/m²), respectively, as previously described (Gong et al., 2019; Chen et al., 2021; Wang et al., 2021).The control group received 10 μL of sterile PBS. The larvae were incubated at 35°C in the dark and monitored daily for survival over 4 days. Larvae showing no movement or no response to gentle touch were recorded as dead. Three independent experiments were performed. Survival data were analyzed using Kaplan–Meier curves and the log-rank test with GraphPad Prism 8.3.0.

#### 2.3.2 Fungal burden analysis

To quantify the fungal burden within infected larvae, an additional set of larvae (n = 20 per group) received identical infection and drug treatments as described above. At 24, 48, 72, and 96 h post-infection, five larvae were randomly collected from each group and sterilized with 75% ethanol, followed by two rinses with sterile PBS to eliminate surface contaminants. Each larva was homogenized in 5 mL of sterile PBS using a tissue grinder. The resulting homogenates were serially diluted ten-fold with PBS, and 10 μL aliquots from appropriate dilutions were spotted onto Sabouraud dextrose agar (SDA) plates. Following incubation at 35°C for 24–48 h, colonies were counted and CFU/larva was calculated as: CFU/larva = colony count × dilution factor × (homogenate volume/plated volume). For plates with no colonies, CFU was set to LOD/2 before log_10_ transformation. The entire procedure was carried out in triplicate across three separate experiments. Statistical evaluation of fungal burden was conducted using one-way ANOVA with Tukey’s *post hoc* test in GraphPad Prism 8.3.0.

#### 2.3.3 Histological examination

Histopathological assessment was undertaken to examine the extent of fungal invasion and host tissue damage in *G. mellonella* larvae. At 48 h following infection and drug administration, five larvae were selected at random from each treatment group and transferred to sterile Petri dishes. The specimens were subsequently placed at -20°C for 10–15 min to achieve immobilization. Frozen sections of 18–22 μm thickness were generated using a cryostat microtome and mounted onto glass slides.

For Periodic Acid-Schiff (PAS) staining, the sections were first hydrated with distilled water, then oxidized with periodic acid for 5–10 min. After rinsing under running tap water, the slides were incubated with Schiff reagent in the dark for 15 min, followed by washing with sodium bisulfite solution. Counterstaining was performed with hematoxylin for 1 min, and the sections were differentiated in acidic ethanol for 10 s before a final tap water rinse. Following staining, the preparations were dehydrated through a graded ethanol series, cleared in xylene, and sealed with neutral mounting medium under coverslips. All sections were examined under the microscope (Nikon Ti-S, Japan) at 4.2 × 10 objective magnification, and representative photomicrographs were captured from each treatment group. Histological analyses were repeated on samples derived from three independent experiments.

### 2.4 Synergistic mechanisms explorations

#### 2.4.1 Rhodamine6G efflux assay

Considering that both Rh6G and FLC act as substrates for drug transporters, the efflux of pharmaceuticals from *C. albicans* was assessed utilizing the Rh6G assay (Soto, 2014). Initially, CA10 cells were produced as a suspension at a concentration of 1×10^5^ CFU/mL and cultivated in YPD liquid medium in a shaking incubator at 200 rpm and 35°C for 18 to 19 hours to promote exponential growth. Subsequent to incubation, cells were collected, subjected to three washes with glucose-free PBS, and resuspended to a concentration of 5×10^6^ CFU/mL. The cells were subsequently exposed to an energy-depleting phase by shaking at 200 rpm and 35°C for 1–2 hours to completely exhaust their energy reserves. Thereafter, Rh6G was incorporated into the deenergized cell suspension to achieve a final concentration of 10 mM. The samples were incubated at 35°C for 1 hour in a shaking incubator to facilitate Rh6G uptake, followed by an instantaneous transfer to an ice-water bath for 30 minutes to terminate further Rh6G absorption. In the experimental group, BTZ was included into the cell suspension at a concentration of 4 µg/mL, whereas the control group consisted solely of Rh6G. The mean fluorescence intensity (MFI) of intracellular Rh6G was quantified at designated time intervals (0, 30, 60, 90, 120, 150, and 180 minutes) utilizing a flow cytometer with excitation and emission wavelengths calibrated to 488 nm and 530 nm, respectively. This experiment was performed in triplicate to guarantee the repeatability and reliability of the results (Jiang et al., 2012; Wang et al., 2021).

#### 2.4.2 Assay for the measurement of reactive oxygen species levels

Exposure of *C. albicans* to specific stimuli induces intracellular ROS accumulation, ultimately triggering programmed cell death through apoptotic pathways (Lee and Lee, 2017). The accumulation of ROS in *C. albicans* CA10 and CA16 (10^6^ CFU/mL) after antifungal treatments was measured using the 2’,7’-dichlorodihydrofluorescein diacetate (DCFH-DA) fluorescence assay. Fungal suspensions were subjected to four experimental conditions for 4 hours at 35°C: (1) untreated control, (2) FLC at 0.25 µg/mL, (3) BTZ at 8 µg/mL, and (4) combination therapy (FLC+BTZ at 0.25 + 8 µg/mL). Post-treatment cells were treated with 40 µM DCFH-DA for 30 minutes in darkness at 35°C. The production of the fluorescent oxidation product (DCF) was assessed via flow cytometry utilizing 488 nm excitation and 530 nm emission wavelengths (Coste et al., 2016). In order to ensure experimental reproducibility, three independent biological replicates were performed.

#### 2.4.3 Hyphal growth assay

*C. albicans* hyphae exhibit critical pathogenic properties including host cell adhesion, tissue invasion, and immune evasion, with morphological transition from yeast to hyphal form being essential for virulence (Naglik et al., 2003). RPMI 1640 medium alone at 37°C is sufficient to induce hyphal formation in *C. albicans* without the need for serum supplementation (Sato et al., 2020). To examine antifungal effects on hyphal development, *C. albicans* strains CA10 and CA16 were used in this assay. Fungal suspensions were adjusted to 2 × 10^5^ CFU/mL in RPMI 1640 medium. Aliquots of 100 µL were distributed onto 96-well plates and subjected to four treatment conditions for 4 hours at 35°C with shaking at 75 rpm: (1) FLC at 0.25 µg/mL, (2) BTZ at 8 µg/mL, (3) an FLC-BTZ combination (0.25 µg/mL + 8 µg/mL), and (4) an untreated control with RPMI 1640. Thereafter, the cell solution was aspirated, and each well was rinsed three times with 200 µL of PBS to eliminate any non-adherent cells. Hyphal formation was quantified using fluorescence microscopy (Nikon Ti-S, Japan) with 40× objective magnification. Three independent biological replicates were performed to ensure experimental reproducibility. The hyphal coverage area was quantified using ImageJ software. Images were converted to 8-bit grayscale, the threshold was set automatically, and the percentage of hyphal-positive area per field (%Area) was calculated for each image. In addition, particle counting was performed as a complementary method to quantify the number of discrete hyphal elements per field. Both methods were applied consistently across all treatment groups. Three images per group from independent experiments were analyzed. Data are presented as mean ± standard deviation (SD).

### 2.5 Statistics

All experiments were conducted in triplicate with independent biological replicates. Statistical analyses were conducted utilizing IBM SPSS Statistics 25 (version 25.0; IBM Corp., Armonk, NY, USA), whereas graphical representations were created with GraphPad Prism 8.3.0 (GraphPad Software, San Diego, CA, USA). The efflux of Rh6G was assessed utilizing the unpaired *t-test*. Comparisons among multiple groups (ROS levels, hyphal coverage area, fungal burden in *G. mellonella*, and PAS-positive area in tissue sections) were analyzed by one-way ANOVA followed by Tukey’s *post hoc* test, after verification of homogeneity of variances. Biofilm CFU enumeration and time-kill curve data were analyzed using two-way repeated-measures ANOVA followed by Dunnett’s *post hoc* test to compare the combination group with each monotherapy group at different time points. Survival data of *G. mellonella* were analyzed using Kaplan–Meier survival curves and the log-rank test. Statistical significance was established at *P* < 0.05 for all experimental comparisons.

## 3 Results

### 3.1 MICs of BTZ alone and in conjunction with FLC against *C. albicans*

The antifungal effectiveness of FLC combined with BTZ against planktonic *C. albicans* was assessed utilizing standardized *in vitro* susceptibility testing. **Table 1** illustrates that BTZ monotherapy exhibited specific antifungal efficacy against six FLC-resistant *C. albicans* isolates (MIC range: 64-128 µg/mL).

Significantly, synergistic effects were detected in FLC-resistant isolates when BTZ (8 µg/mL) was administered in conjunction with FLC, decreasing the FLC MIC from >128 µg/mL to 0.25 µg/mL (FICI: 0.064-0.127). For FLC-sensitive *C. albicans* isolates, combined therapy with BTZ resulted in a 32-64-fold decrease in BTZ MIC values (from ≥128 µg/mL to 2–4 µg/mL), while maintaining sensitivity to FLC (MIC = 0.25µg/mL). Despite the fact that FICI values greater than 0.5 indicate an absence of pharmacodynamic synergy, this combination remains of interest.

### 3.2 sMICs of BTZ in conjunction with FLC against biofilms of *C. albicans*

The BTZ-FLC combination demonstrated synergistic antifungal efficacy against *C. albicans* planktonic cells. The susceptibility of biofilms was further assessed using two fluconazole-susceptible (CA4, CA8) and two fluconazole-resistant (CA10, CA16) clinical isolates by modified biofilm minimum inhibitory concentration (sMIC) experiments (**Table 2**). The FLC-BTZ combination exhibited synergistic antifungal efficacy against immature *C. albicans* during the early stages of biofilm formation (4 and 8 h) in both FLC-susceptible and resistant strains, with FICI values below 0.5. This combinatorial regimen decreased the sMIC of BTZ from ≥128 µg/mL to 8-16 µg/mL and FLC from ≥128 µg/mL to 0.25 µg/mL. Nonetheless, no synergism was seen in biofilms that had been established for over 12 hours, with FICI values exceeding 0.5.

**Table 2:** Drug interactions of bortezomib (BTZ) and fluconazole (FLC) against biofilms of *Candida albicans*.

Strains^a^	Time^b^	sMIC_80_ (μg/mL)^c^				FICI^c^	Interaction^d^
		Alone		Combined			
		FLC	BTZ	FLC	BTZ		
CA10(R)	4h	>128	128	0.25	8	0.0644	SYN
	8h	>128	128	0.25	16	0.1269	SYN
	12h	>128	128	0.25	128	1.0019	NI
	24h	>128	128	0.25	128	1.0019	NI
CA16(R)	4h	>128	128	0.25	8	0.0644	SYN
	8h	>128	>128	0.25	16	0.1269	SYN
	12h	>128	128	0.25	128	1.0019	NI
	24h	>128	128	0.25	128	1.0019	NI
CA4(S)	4h	>128	>128	0.25	8	0.0644	SYN
	8h	>128	>128	0.25	16	0.1269	SYN
	12h	>128	128	0.25	128	1.0019	NI
	24h	>128	128	0.25	128	1.0019	NI
CA8(S)	4h	>128	>128	0.25	8	0.0644	SYN
	8h	>128	>128	0.25	16	0.1269	SYN
	12h	>128	128	0.25	128	1.0019	NI
	24h	>128	128	0.25	128	1.0019	NI

^a^ CA, Candida albicans.

^b^ Time indicates the incubation period of biofilm formation.

^c^ FICI, fractional inhibitory concentration index; sMIC was defined as 80% inhibition of biofilm metabolic activity compared with growth control group.

sMIC, values and FICIs are shown as the median of three independent experiments.

^d^ SYN, synergism; NI, no interaction.

### 3.3 CFU enumeration of biofilm cells

To further evaluate the anti-biofilm activity of the FLC+BTZ combination, CFU enumeration was performed against pre-formed biofilms of *C. albicans* CA10 at an initial inoculum of 10^6^ CFU/mL, following treatment for 4, 8, 12, and 24 h. Two combination concentrations (0.25 + 8 and 0.25 + 16) were tested. The 0.25 + 8 combination showed only a transient reduction in viable cell counts at 4, 8, and 12 h, but this effect was not sustained at 24 h (**Supplementary Figure 1**). In contrast, the 0.25 + 16 combination exhibited a sustained reduction at all tested time points (4 h: *P* = 0.0063; 8 h: *P* = 0.0025; 12 h: *P* = 0.0057; 24 h: *P* = 0.0392) (**Figure 1**). Two-way repeated-measures ANOVA revealed significant main effects and interaction for both datasets (*P* < 0.0001 for each). These results indicate a concentration-dependent inhibitory effect on biofilm viability, with the higher concentration (0.25 + 16) showing more sustained activity through 24 h.

**Figure 1:**
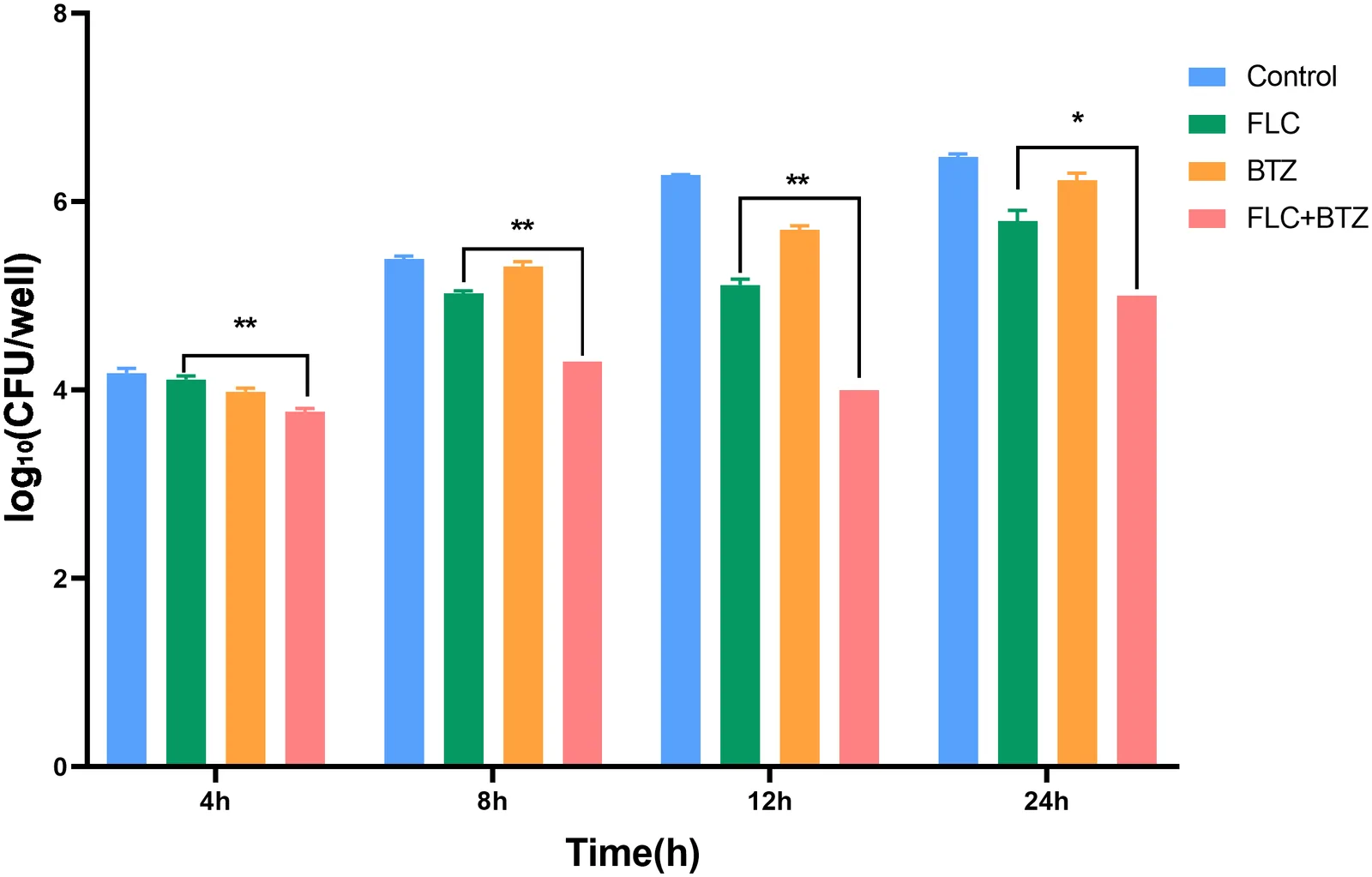
Anti-biofilm activity determined by CFU enumeration. Biofilms of *C. albicans* CA10 (1 × 10^6^ CFU/mL) were treated with FLC (0.25 µg/mL), BTZ (16 µg/mL), or their combinations (0.25 + 16) for 4, 8, 12, and 24 h. Viable cells were quantified by CFU counting. Data are presented as mean ± SD from three independent experiments. *P < 0.05, **P < 0.01, (two-way repeated-measures ANOVA with Dunnett’s *post hoc* test).

### 3.4 Time-kill kinetics against planktonic cells

The time-kill kinetics of the FLC+BTZ combination against planktonic cells of *C. albicans* CA10 were evaluated over 48 h at an initial inoculum of 10^5^ CFU/mL. The combination of FLC and BTZ at 0.25 + 8 exhibited delayed inhibitory activity. Compared with FLC alone, the combination resulted in a significant reduction in viable cell counts at 24 h (*P =* 0.0490) and 48 h (*P =* 0.0045). No significant differences were observed at 6 h (*P* = 0.9980) or 12 h (*P* = 0.2122) (**Figure 2**). Two-way repeated-measures ANOVA revealed significant main effects and interaction (*P* < 0.0001 for each), with significant reductions at 24 h and 48 h vs. FLC alone (Dunnett’s test).These results indicate that the FLC+BTZ combination exerts a delayed but sustained inhibitory effect against planktonic *C. albicans* cells, with efficacy becoming apparent at 24 h and persisting through 48 h.

**Figure 2:**
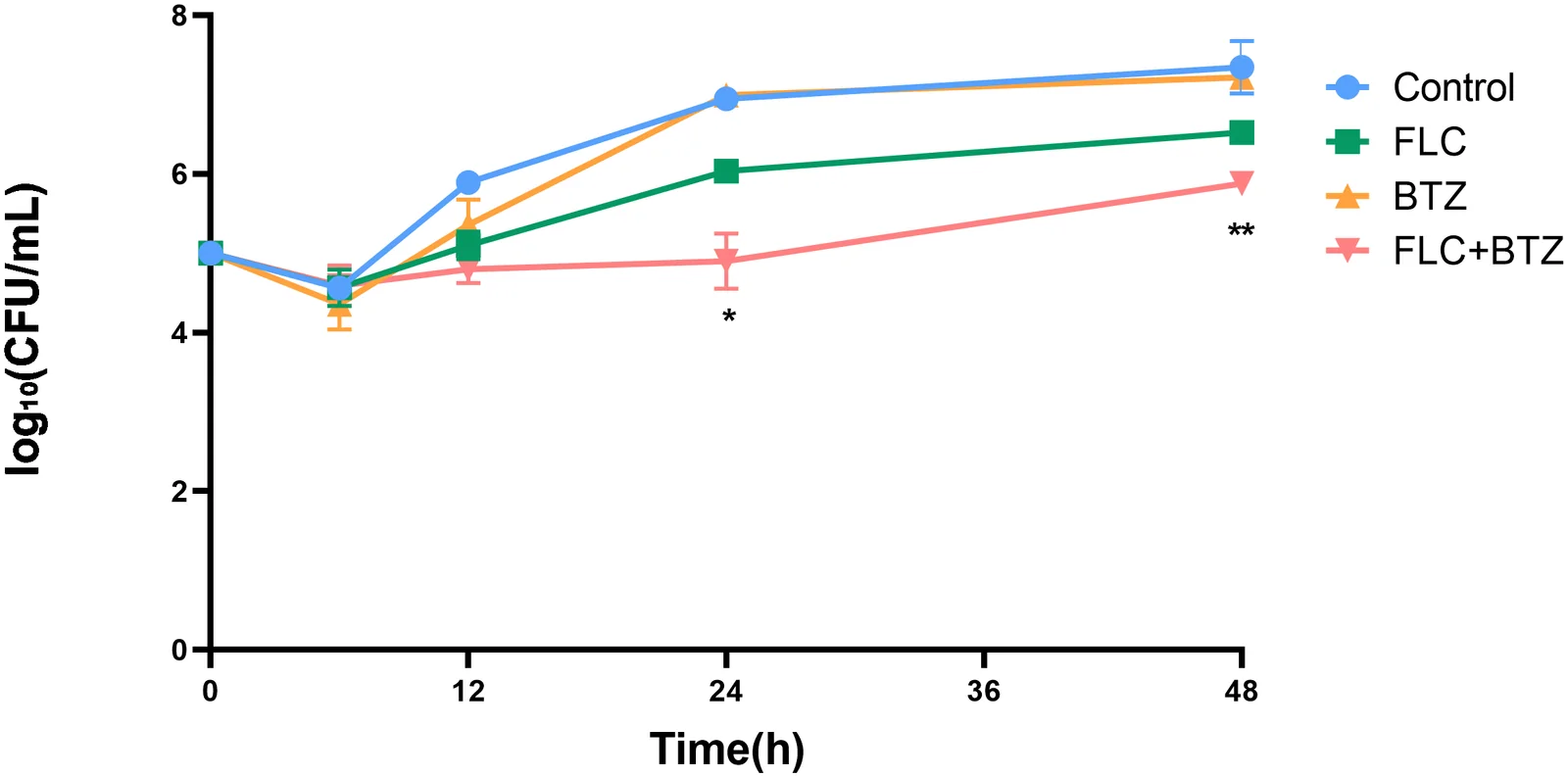
Time-kill kinetics against planktonic *C. albicans*. *C. albicans* CA10 (1 × 10^5^ CFU/mL) was treated with FLC (0.25 µg/mL), BTZ (8 µg/mL), or FLC+BTZ (0.25 + 8 µg/mL). Viable counts were determined at 0, 6, 12, 24, and 48 h. Data are presented as mean ± SD from three independent experiments. *P < 0.05, **P < 0.01 (two-way repeated-measures ANOVA with Dunnett’s *post hoc* test).

### 3.5 Antifungal effects of BTZ and FLC against *C. albicans in vivo*

#### 3.5.1 Survival assays

Kaplan-Meier survival analysis was performed to evaluate the *in vivo* antifungal efficacy of the FLC+BTZ combination against *C. albicans* infection in *G. mellonella* larvae. As shown in **Figure 3**, the median survival time of larvae in the control group was 2 days, whereas treatment with FLC+BTZ (160 + 4 μg/mL) prolonged larval survival to 3 days. The Log-rank test revealed a statistically significant difference between the survival curves of the control and FLC+BTZ groups (*P* = 0.0011). Compared with FLC monotherapy, the combination also significantly improved survival (P = 0.0100). These results indicate that the FLC+BTZ combination improves larval survival compared with both the control and FLC monotherapy groups in the *G. mellonella* infection model.

**Figure 3:**
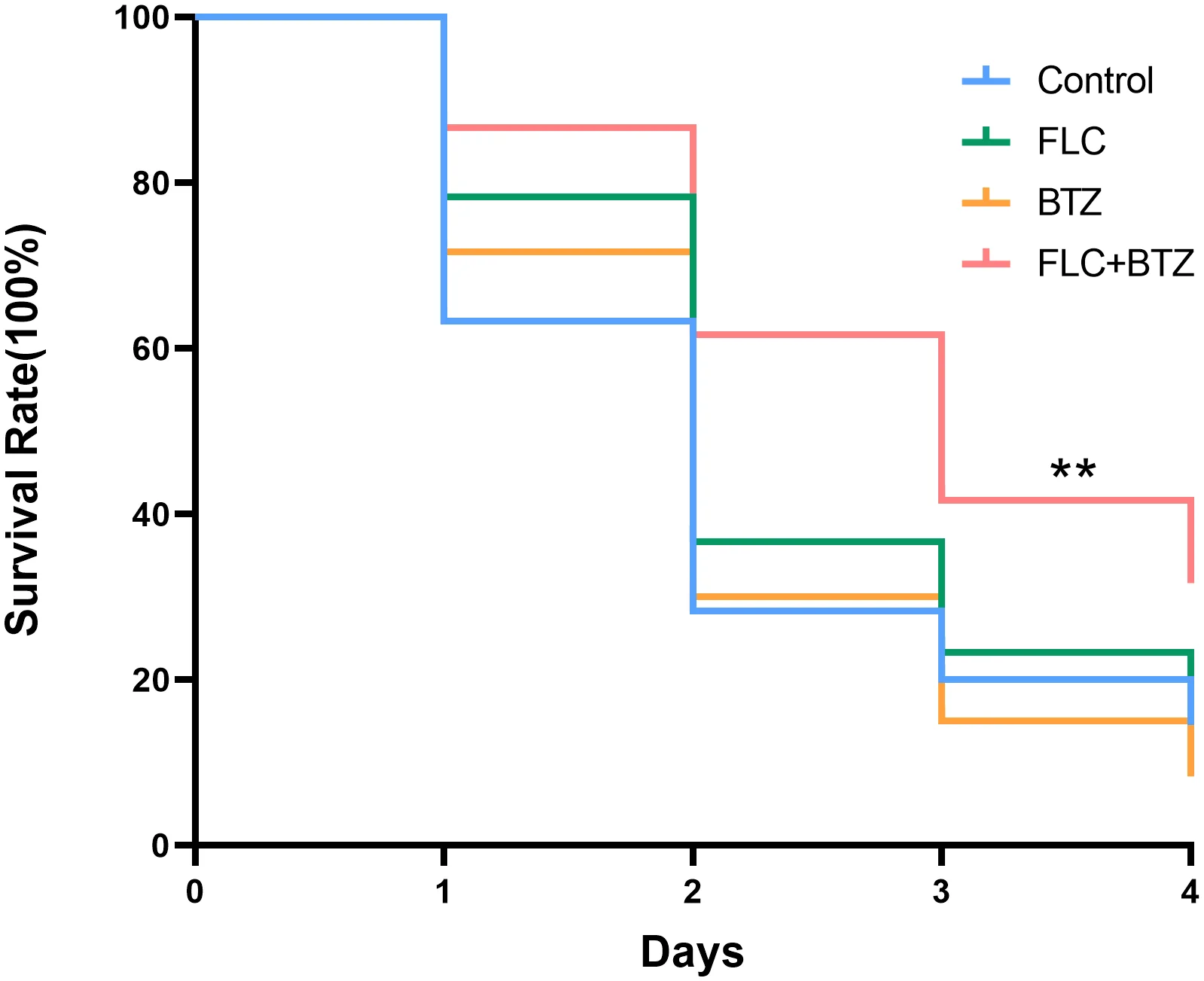
*In vivo* antifungal efficacy in the *Galleria mellonella* infection model (survival assay). Kaplan–Meier survival curves of larvae infected with *C. albicans* CA10 (5 × 10^8^ CFU/mL, 10 μL per larva) and treated with PBS (control), FLC (160 μg/mL), BTZ (4 μg/mL), or FLC+BTZ (160 + 4 μg/mL). Survival was monitored daily for 4 days. Data are representative of three independent experiments (n = 20 larvae per group per experiment). **P < 0.01 (Log-rank test).

#### 3.5.2 Determination of fungal burden

Fungal burden analysis revealed that the combination of FLC and BTZ (160 + 4 μg/mL) significantly reduced fungal burden compared with both the control and FLC monotherapy groups at all tested time points (*P* < 0.05 for each), with the most pronounced reduction observed at 72 h (*P* = 0.0012 vs. control; *P* = 0.0018 vs. FLC) (**Figure 4**). Two-way repeated-measures ANOVA revealed significant main effects of treatment and time, as well as a significant treatment × time interaction (p < 0.0001 for each). These results are consistent with the survival data.

**Figure 4:**
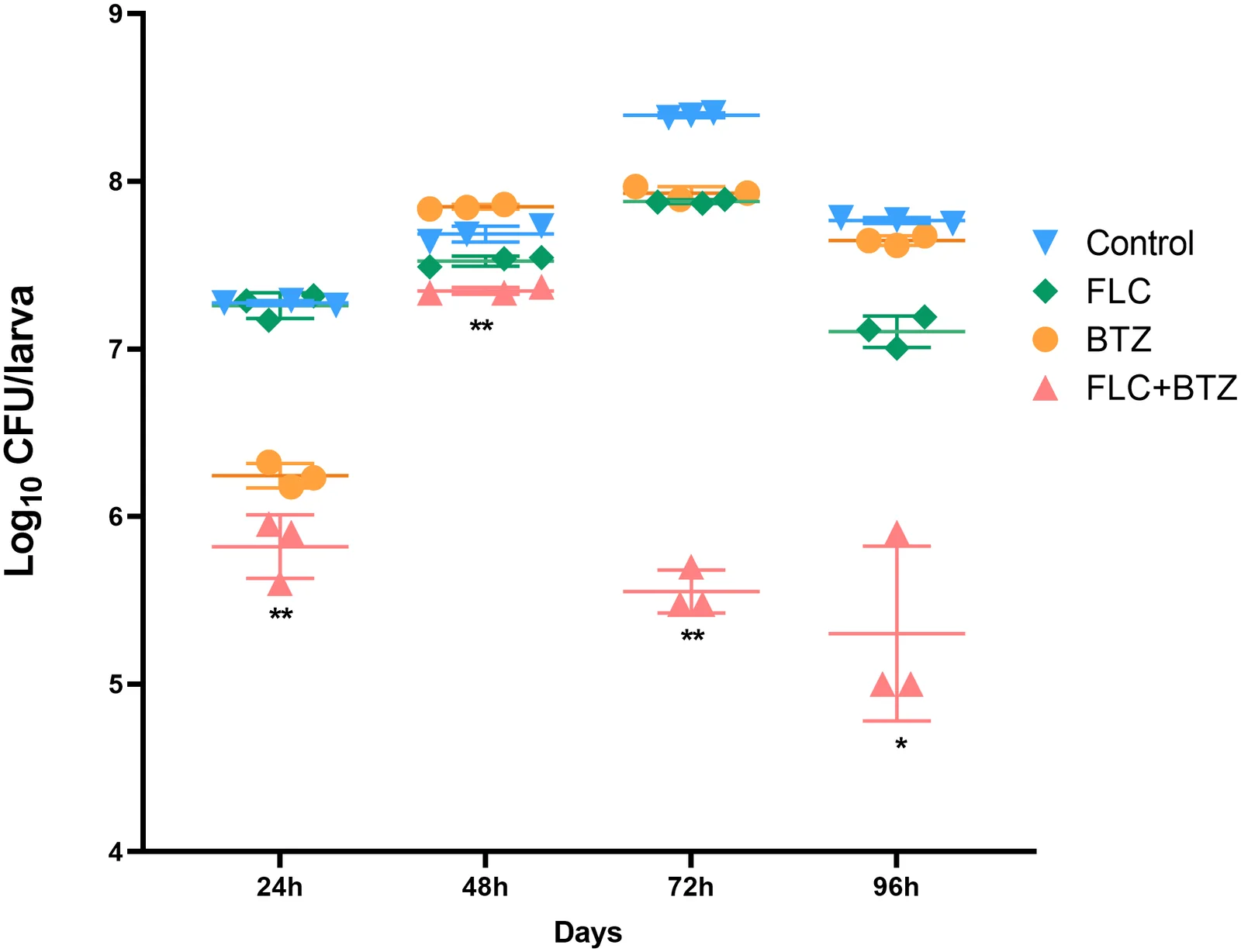
Fungal burden in *Galleria mellonella* larvae infected with *C. albicans* CA10. Larvae were treated with PBS (control), FLC (160 μg/mL), BTZ (4 μg/mL), or FLC+BTZ (160 + 4 μg/mL). Fungal burden was determined at 24, 48, 72, and 96 h post-infection. Data are presented as mean ± SD from three independent experiments (n = 5 larvae per group per time point). *P < 0.05, **P < 0.01 (two-way repeated-measures ANOVA with Dunnett’s *post hoc* test).

#### 3.5.3 Histological study

Histopathological examination of PAS-stained tissue sections at 48 h post-infection revealed abundant fungal elements and melanized nodules in the control group, indicating severe fungal colonization and tissue damage (**Figure 5A**). Similar pathological features were observed in the FLC monotherapy group. In contrast, larvae treated with the FLC+BTZ combination showed fewer fungal elements and melanized nodules, with relatively preserved tissue architecture (**Figure 5A**). ImageJ-based quantification of PAS-positive area (%Area) showed that the fungal coverage area in the combination group was significantly lower than that in the FLC monotherapy group (P = 0.0014) (**Figure 5B**). These results are consistent with the survival and fungal burden data.

**Figure 5:**
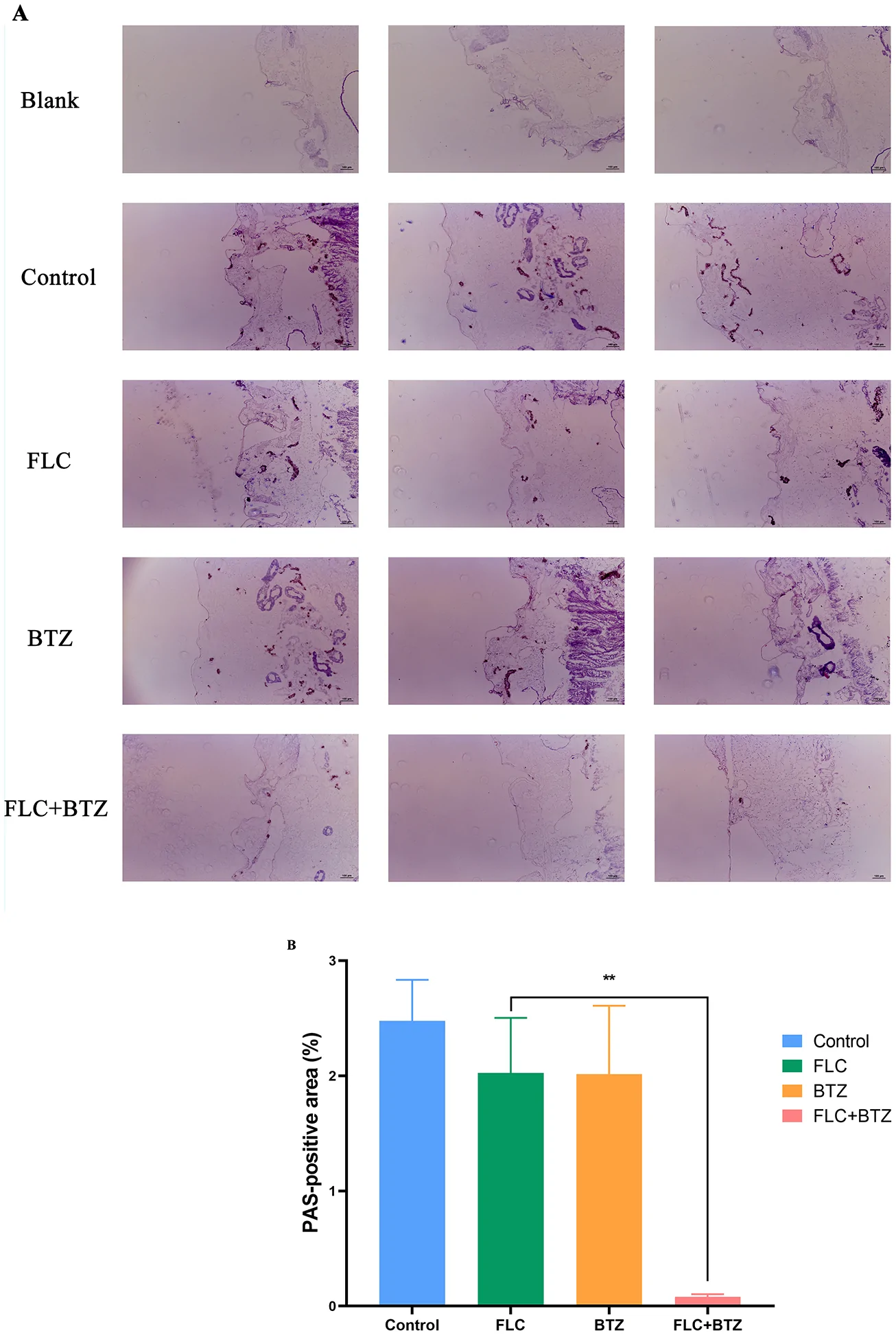
Histopathology of *Galleria mellonella* infected with *C. albicans*. **(A)**The concentration of yeast cells was 5 × 10^8^ CFU/mL (10 μL per larva). Treatments consisted of PBS (control), FLC (160 μg/mL), BTZ (4 μg/mL), and the combination of FLC and BTZ (160 + 4 μg/mL). PAS staining was performed at 48 h post-infection, and representative images were captured from each group. Melanized nodules, which appeared as black/brown aggregates, were the mixtures of yeast cells and filaments. Scale bar = 100 μm. **(B)** ImageJ-based quantification of PAS-positive area (%Area). Data are presented as mean ± SD from three independent experiments. **P< 0.01 (one-way ANOVA with Sidak’s *post hoc* test).

### 3.6 Exploration of mechanisms

#### 3.6.1 Rh6G efflux assay

Given that both Rh6G and FLC function as substrates for the ABC transporters Cdr1p and Cdr2p in *C. albicans*, Rh6G was utilized as a confirmed fluorescent surrogate to observe drug transporter activity in real-time efflux tests. In the initial 60 minutes, the fluorescence intensity of both the control and BTZ (4 µg/mL) groups exhibited a progressive decline, however no significant difference was observed. After 90 minutes, the fluorescence intensity in the BTZ group was markedly elevated compared to the control group (90–120 minutes, *P* < 0.01; 150–180 minutes, *P* < 0.05), as illustrated in **Figure 6**. In conclusion, these findings indicate that the combination of BTZ with FLC exerts a synergistic impact against FLC-resistant *C. albicans* through the suppression of the efflux pump.

**Figure 6:**
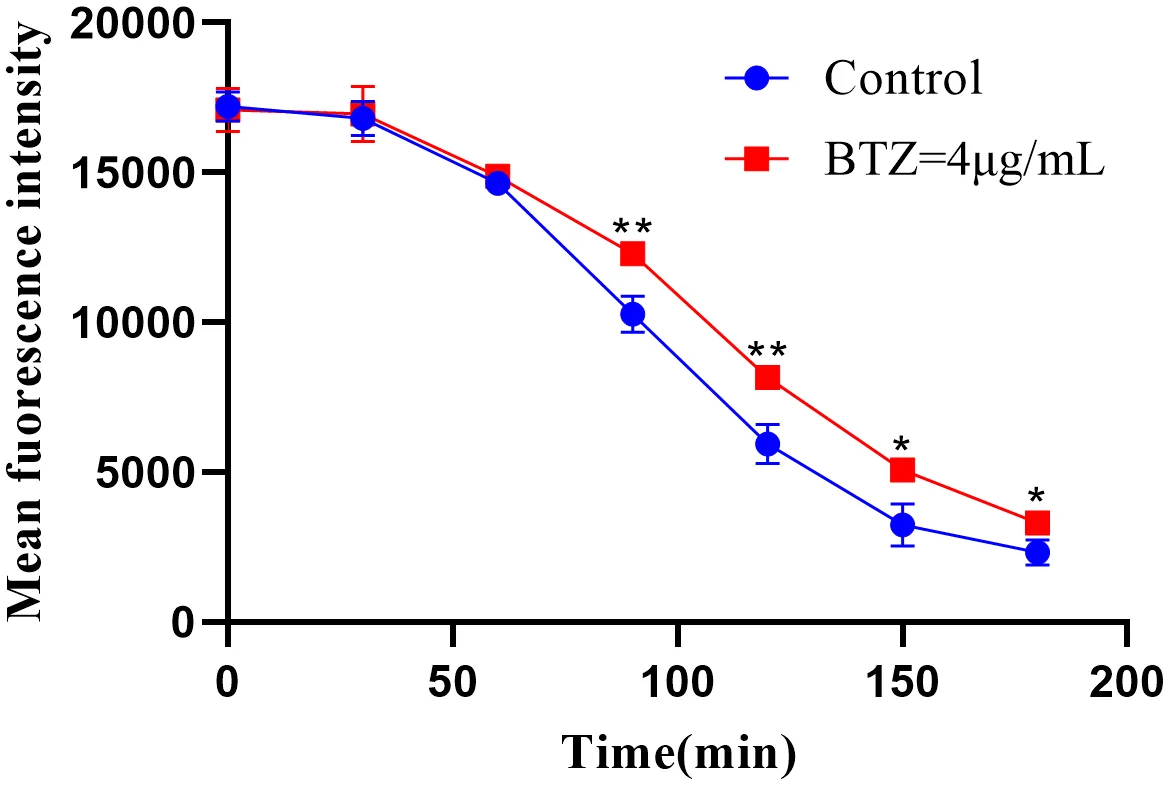
Effect of bortezomib on the efflux of Rh6G in *C. albicans*. The concentration of CA10 was 5 × 10^6^ colony-forming units/mL. The efflux of Rh6G in the absence and presence of bortezomib (BTZ; 4 μg/mL) was determined by a flow cytometer. Mean fluorescence intensity represent the intracellular Rh6G in *C. albicans*. The data came from the means of three independent experiments. **P < 0.01, *P < 0.05.

#### 3.6.2 Efficacy of BTZ and FLC on reactive oxygen species generation in *C. albicans*

A defining characteristic of apoptosis in *C. albicans* is the intracellular buildup of ROS, which serves as an initial trigger for programmed cell death. To evaluate whether this mechanism is conserved across strains, we examined ROS production in both CA10 and CA16. As shown in **Figure 7A**, in CA10, treatment with FLC (0.25 µg/mL), BTZ (8 µg/mL), and FLC+BTZ (0.25 + 8 µg/mL) increased the mean fluorescence intensity (MFI) by approximately 4%, 28%, and 80%, respectively, compared with the control group. In CA16, the combination of FLC+BTZ (0.25 + 8 µg/mL) also significantly elevated ROS levels compared with FLC alone (*P* < 0.01)(**Figure 7B**). These results indicate that the FLC+BTZ combination promotes intracellular ROS accumulation in both strains, suggesting that the antifungal activity is associated with ROS accumulation.

**Figure 7:**
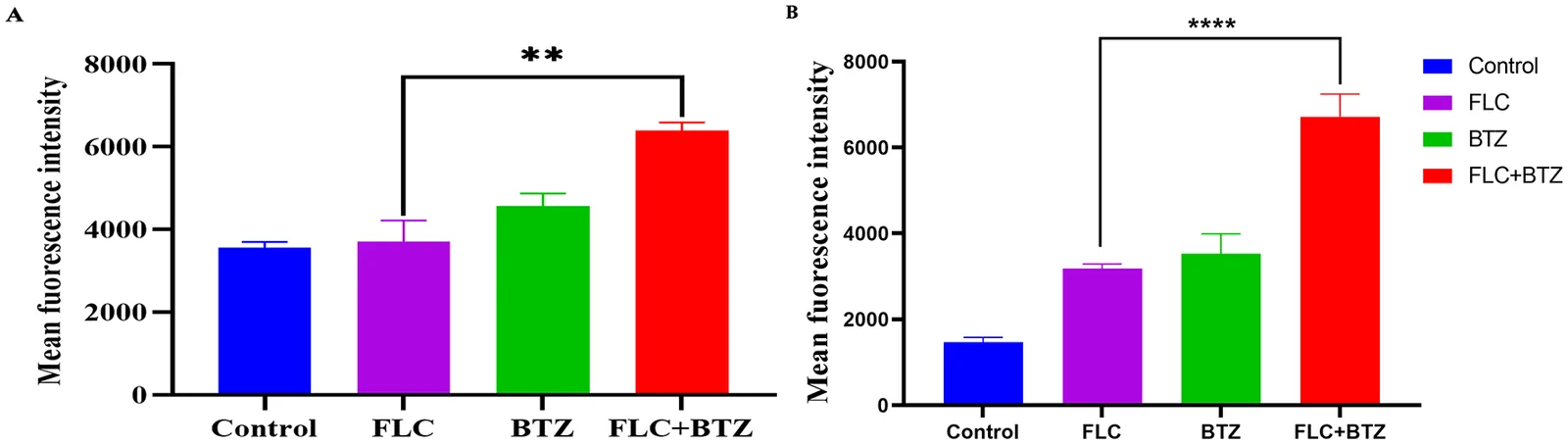
Effect of different drugs on cellular levels of reactive oxygen Species (ROS). **(A)** CA10 and **(B)** CA16 cells (1 × 10^6^ CFU/mL)*C. albicans* were treated with diverse concentrations of bortezomib (BTZ; 8 μg/mL), fluconazole (FLC; 0.25 µg/mL) and FLC (0.25 μg/mL) + BTZ (8 μg/mL) in combination for 4 h, and the intracellular levels of reactive oxygen species were measured by DCFH-DA assay. The experiment was repeated on three independent occasions. The levels of the ROS were analyzed by one-way ANOVA and *Post Hoc* (LSD test). **P < 0.01, ****P < 0.0001.

#### 3.6.3 Efficacy of BTZ and FLC on *C. albicans* hyphal development

Previous study indicates that the yeast-to-hypha transition is a critical virulence element linked to the pathogenesis of *C. albicans* (Sudbery et al., 2004; Modrzewska and Kurnatowski, 2013; Li et al., 2019). To evaluate the effect of the FLC+BTZ combination on hyphal formation, we examined hyphal development in CA10 and CA16 strains. Microscopic images revealed distinct hyphal morphogenesis patterns among treatment groups (**Figures 8A, B**). The control and FLC monotherapy groups exhibited dense, elongated hyphal networks, whereas the FLC+BTZ combination showed reduced hyphal development. ImageJ-based quantification of hyphal coverage area (%Area) confirmed a significant reduction in the combination group compared with the FLC group in both CA10 and CA16 (*P* < 0.0001 for each) (**Figures 8C, D**).These results suggest that the FLC+BTZ combination is associated with the inhibition of hyphal formation, which may contribute to its antifungal activity.

**Figure 8:**
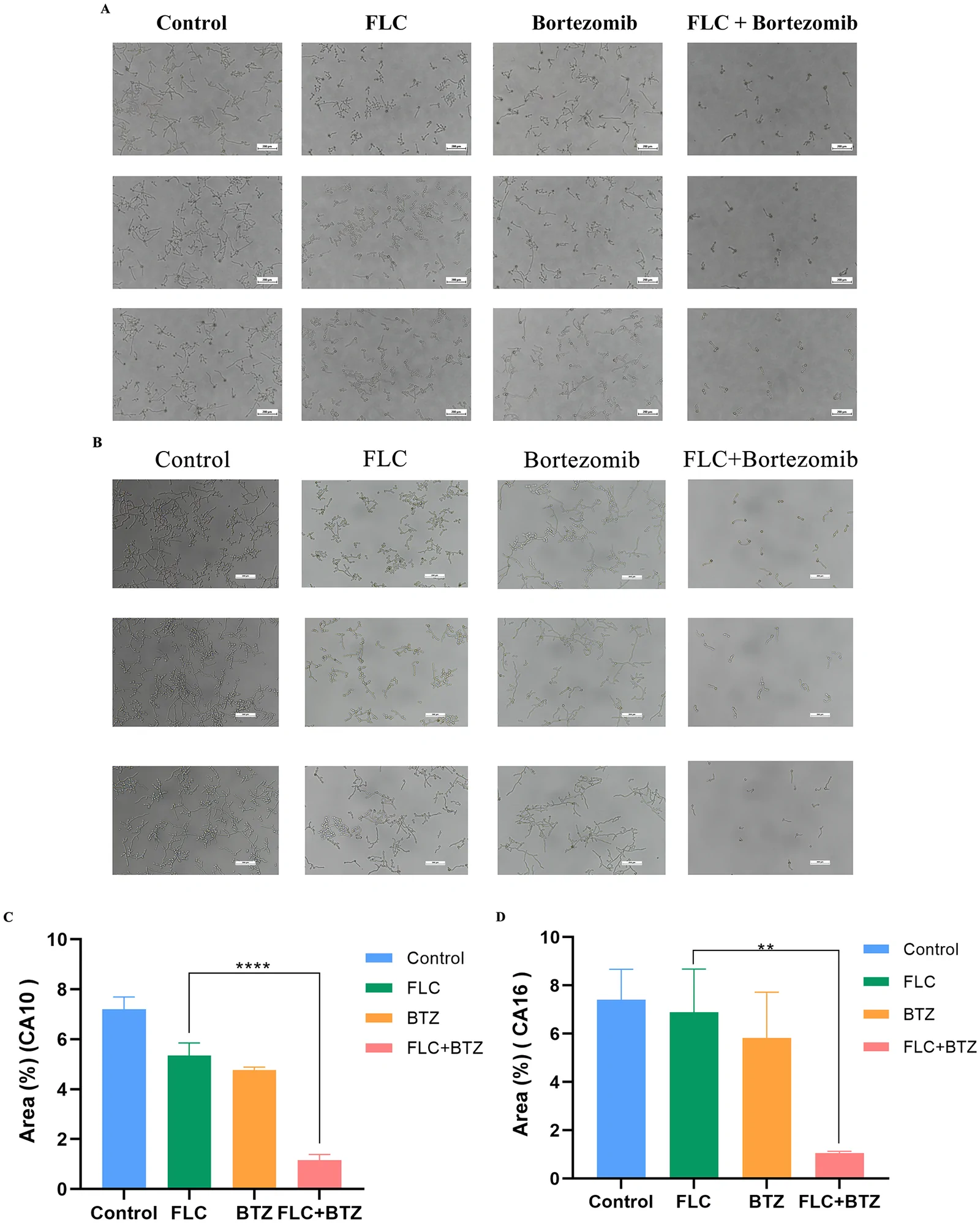
Effect of different drugs on *C. albicans* hyphal growth. **(A)** Representative microscopic images of hyphal morphology in CA10. **(B)** Representative microscopic images of hyphal morphology in CA16. Cells (2 × 10^5^ CFU/mL) were incubated in RPMI 1640 medium at 37 °C for 4 h with FLC (0.25 µg/mL), BTZ (8 μg/mL), or FLC+BTZ (0.25 + 8 μg/mL). Scale bar = 200 μm. **(C)** ImageJ-based quantification of hyphal coverage area (%Area) in CA10. **(D)** ImageJ-based quantification of hyphal coverage area (%Area) in CA16. Data are presented as mean ± SD from three independent experiments. ****P < 0.0001 vs. FLC group (CA10), **P < 0.01 vs. FLC group (CA16) (one-way ANOVA with Sidak’s *post hoc* test).

## 4 Discussion

The escalating mortality associated with invasive *C. albicans* infections has established this pathogen as a critical impediment to clinical recovery, particularly in immunocompromised populations (Cornely et al., 2017). Key predisposing factors include medical interventions (broad-spectrum antimicrobial overuse, immunosuppressive therapies, indwelling catheters) and emerging comorbidities (solid-organ transplantation sequelae, radiotherapy-induced immunosuppression, HIV/AIDS progression), which collectively compromise host defense mechanisms and create permissive niches for fungal dissemination (Alangaden, 2011). Recent studies have indicated that repurposing pharmaceuticals by combining non-antifungal agents with conventional antifungals may offer a viable solution to counteract *Candida* resistance mechanisms (Feng et al., 2021). Emerging evidence demonstrates the synergistic efficacy of ribavirin (an inhibitor of viral RNA synthesis), ambroxol hydrochloride (a mucolytic agent), lonidamine (a mitochondrial modulator), and benserazide (an aromatic amino acid decarboxylase inhibitor) with FLC against azole-resistant *C. albicans* through different sensitization mechanisms, including biofilm matrix disruption and drug efflux modulation (Li et al., 2017; Chen et al., 2020; Zhang et al., 2020; Chen et al., 2021). In support of this concept, recent studies have shown that antifungal combination or alternation strategies can effectively reduce drug-tolerant populations, thereby minimizing the development of resistance (Cai et al., 2026).

BTZ is a proteasome inhibitor clinically employed in multiple myeloma therapy (e.g., melphalan/prednisone regimens) (Mateos et al., 2020),which has also been reported to exhibit antimicrobial potential (Kaneko et al., 2013; Supuran, 2016).Notably, existing investigations have exclusively evaluated BTZ-FLC synergy against the standard strain *C. albicans* SC5314 (Kaneko et al., 2013), while critical knowledge gaps persist regarding: (1) the effectiveness against fluconazole-resistant clinical *C. albicans* isolates; (2) the effectiveness against *C. albicans* biofilms; (3) the molecular foundation of the anti-*C. albicans* activity. This research methodically assessed the combined efficacy of BTZ and FLC against *C. albicans*. The BTZ monotherapy group exhibited antifungal efficacy (MIC = 64 µg/mL or >128 µg/mL), and when administered in conjunction with FLC (BTZ = 8 µg/mL), the MIC of FLC decreased from >128 µg/mL to 0.25 µg/mL (FICI: 0.064-0.127), indicating substantial synergistic effects on FLC-resistant isolates. Although classical synergism was absent in FLC-sensitive strains, BTZ significantly enhanced FLC efficacy through concentration-dependent interactions. The lack of synergy in FLC-susceptible strains can be attributed to several factors. First, the primary mechanism of BTZ-mediated synergism in resistant strains is the inhibition of efflux pump activity, which reduces the intracellular extrusion of FLC. In FLC-susceptible strains, the baseline expression of these efflux pumps is already low, and the intrinsic susceptibility to FLC is high (MIC ≤ 0.5 μg/mL), limiting the scope for BTZ to exert additional chemosensitizing effects. Second, the synergistic effect of azole combinations often depends on the presence of active resistance mechanisms. Consistent with this, previous studies have shown that various compounds—including ribavirin (Zhang et al., 2020), benserazide hydrochloride (Chen et al., 2021), and panobinostat (Su et al., 2020)—exhibit synergistic antifungal activity exclusively against FLC-resistant *C. albicans* isolates, with no such synergy observed in susceptible strains. Third, the FLC MIC values in susceptible strains are already at the lower limit of detection, creating a “ceiling effect” that makes it difficult to observe further reductions through combination therapy. These findings collectively support the notion that the synergistic interaction is dependent on the presence of resistance mechanisms, and that FLC-resistant infections represent the more clinically challenging scenario and are the primary target for this combination strategy.

The development of biofilms is a crucial determinant of the pathogenicity of *C. albicans*, facilitating the organism’s colonization of medical devices and human tissues (Nobile and Johnson, 2015). The biofilm-forming *C. albicans* demonstrate significantly greater resistance to antimicrobial agents and immunological defenses than planktonic cells (Heitman et al., 2012; Mayer et al., 2014). Our biofilm susceptibility analysis (**Table 2**) revealed a time-dependent synergistic activity of BTZ-FLC combinations. For biofilms at the early stages of formation (4–8 h) strong synergistic effects were observed (FICI < 0.5), with FLC sMIC decreasing from >128 µg/mL to 0.25 µg/mL at BTZ concentrations of 8-16 µg/mL. No synergism was detected in biofilms that had been established for over 12 hours. These results indicate that the FLC+BTZ combination exhibits anti-biofilm activity against *C. albicans* during the early stages of biofilm formation (within 12 h). However, its activity against mature (24 h) biofilms is limited, representing a limitation of the current study and warrants further investigation to optimize the combination for clinical applications. The CFU enumeration assay demonstrated that the FLC+BTZ combination reduced biofilm viability in a concentration-dependent manner. The lower concentration (0.25+8 μg/mL) showed only a transient reduction (4–12 h), whereas the higher concentration (0.25+16 μg/mL) sustained this effect through 24 h. This suggests that a threshold BTZ concentration is required for durable activity against high-density biofilms. The loss of effect at the lower concentration at 24 h may reflect limited drug penetration into mature biofilms or reduced susceptibility of cells within the biofilm matrix, possibly due to the protective role of the extracellular matrix. These findings are consistent with previous reports that biofilm-associated cells exhibit decreased susceptibility to antifungal agents (Ramage et al., 2005; Uppuluri et al., 2008). The time-kill assay revealed that the FLC+BTZ combination (0.25+8 μg/mL) exhibited a delayed but sustained inhibitory effect against planktonic *C. albicans* cells, with significant reductions observed at 24 h and 48 h but not at 6 h or 12 h. This delayed onset may be attributed to the high initial inoculum (10^5^ CFU/mL), requiring prolonged drug exposure to achieve detectable killing—consistent with the inoculum effect described in antifungal pharmacology. Notably, the combination showed a different temporal profile in biofilms (early but transient activity at 0.25+8 μg/mL), suggesting that the susceptibility to the combination may differ between planktonic and biofilm cells.

To determine whether the synergistic antifungal effect observed *in vitro* could be reproduced *in vivo*, we employed the *G. mellonella* infection model, which offers clear advantages in ethics, logistics, and cost for rapid evaluation of antimicrobial efficacy (Borman, 2018; Gago et al., 2014).Survival assays revealed that the FLC+BTZ combination significantly improved larval survival compared with FLC alone. The median survival time was extended from 2 days (FLC) to 3 days (combination), with a significant difference between the two groups (*P* = 0.0100) and a hazard ratio of 0.6433 (95% CI: 0.4264 to 0.9706). These results indicate that BTZ enhances the *in vivo* efficacy of FLC. Fungal burden analysis further supported the synergistic effect, as the combination significantly reduced viable fungal counts compared with FLC alone at all tested time points (24–96 h), with the greatest reduction observed at 72 h (approximately 2.33 log_10_ CFU/larva).Histological examination of PAS-stained sections showed extensive fungal elements and melanized nodules in the control and FLC groups, indicating severe tissue damage. In contrast, the combination group exhibited fewer fungal elements and melanized nodules, with relatively preserved tissue architecture. ImageJ-based quantification confirmed a significant reduction in PAS-positive area in the combination group compared with the FLC group (*P* = 0.0014).

Collectively, these *in vivo* data demonstrate that the FLC+BTZ combination effectively reduces fungal burden and tissue damage caused by resistant *C. albicans* in *G. mellonella* larvae, consistent with the *in vitro* findings.

We further investigated the mechanistic basis of BTZ’s antifungal activity against *C. albicans* in monotherapy and combination groups. Considering the recognized function of efflux pumps in azole resistance, we employed Rh6G, a fluorescent efflux pump substrate, to assess BTZ-mediated transporter modulation. This methodological approach enabled precise evaluation of drug-efflux interactions without direct FLC interference (Pina-Vaz et al., 2005; Soto, 2014). Our data demonstrate that BTZ compromises efflux pump functionality in *C. albicans* CA10, leading to enhanced intracellular FLC retention (**Figure 6**). This efflux inhibition constitutes a principal mechanism underlying the observed BTZ-FLC synergistic interaction. While this experiment was performed using CA10 as the representative strain, the consistency of the synergistic mechanism was further validated in an additional resistant isolate, CA16, through ROS and hyphal inhibition assays. We acknowledge that extending the Rh6G efflux assay to additional resistant strains would further strengthen the generalizability of these findings, and this represents a direction for future investigation.

It has been demonstrated that elevated levels of ROS are conducive to apoptosis. Accumulation of these species has been observed to disrupt oxidative damage to important intracellular biomolecules, thereby ultimately triggering apoptosis (Chen et al., 2013; Tian et al., 2017). Previous investigations have indicated that the combination of non-antifungals with FLC induces anti-*C. albicans* action via promoting intracellular ROS generation (Gong et al., 2019; Wang et al., 2021). In the present study, treatment with BTZ alone increased ROS levels, and the combination with FLC resulted in a more pronounced elevation compared with FLC alone (**Figure 7**). These observations suggest that ROS accumulation may contribute to the antifungal activity of the FLC+BTZ combination, although the precise mechanisms remain to be further investigated.

The resistance of *C. albicans* to most antifungal agents is largely attributed to the development of hyphae (Sudbery, 2011; Noble et al., 2016). Consequently, it is essential to examine the potential inhibitory impact of pharmaceuticals on morphological transformation.

According to the results of biofilm formation inhibition, the hyphae in the FLC (0.25 µg/mL) and BTZ (8 µg/mL) monotherapy group displayed an elongated and dense morphology, whereas the hyphae in the drug combination group showed a decrease in length and quantity (refer to **Figure 8**). The results indicate that the combination of BTZ and FLC may effectively impede the morphological change of *C. albicans*. The ability of *C. albicans* to transition from yeast to hyphal form is considered an important virulence factor contributing to its pathogenicity (Sudbery et al., 2004). In the present study, microscopic examination revealed that treatment with FLC (0.25 µg/mL) or BTZ (8 µg/mL) alone resulted in dense and elongated hyphal networks, whereas the FLC+BTZ combination (0.25 + 8 μg/mL) was associated with reduced hyphal length and density (**Figures 8A, B**). ImageJ-based quantification further confirmed a significant reduction in hyphal coverage area in the combination group compared with FLC alone (**Figures 8C, D**). The suppression of yeast-to-hyphal transition may contribute to the reduced virulence potential of *C. albicans* (Zhao et al., 2026).

Several limitations should be acknowledged. First, the anti-biofilm activity was limited to the early stages of biofilm formation (within 12 h), with reduced efficacy against mature (24 h) biofilms. Second, the mechanistic studies were performed using resistant strains only, and the conclusions may not be generalizable to susceptible isolates; we also lacked direct evidence for apoptosis or hyphal gene regulation. Third, the *G. mellonella* model does not fully replicate mammalian infections. Fourth, the BTZ concentrations effective *in vitro* (8–16 μg/mL) are higher than those achieved systemically in clinical use, and the safety of this combination in mammals requires further evaluation. Fifth, while light microscopy with ImageJ quantification provided sufficient evidence for hyphal inhibition, advanced imaging techniques such as CLSM and SEM represent promising future directions. Sixth, while the XTT and CFU assays provided complementary evidence for anti-biofilm activity, we did not perform crystal violet staining to quantify total biofilm biomass, which remains a limitation of the current study. Future studies incorporating mammalian models, gene expression analyses, and toxicological evaluations would help to further elucidate the mechanisms and translational potential of this combination.

## 5 Conclusions

In conclusion, this study demonstrates that BTZ enhances the antifungal activity of FLC against drug-resistant *C. albicans* across multiple strains. The combination exhibited synergistic effects against both planktonic cells and biofilms *in vitro*, and these findings were supported by *in vivo* experiments using the *G. mellonella* infection model, where the combination improved larval survival, reduced fungal burden, and alleviated tissue damage compared with FLC monotherapy. Mechanistic studies indicate that this synergy may involve the inhibition of efflux pumps, enhancement of ROS-mediated oxidative stress, and suppression of the yeast-to-hyphae morphological transition. Although the precise molecular mechanisms require further elucidation, these findings provide a basis for the potential application of BTZ as an adjuvant to FLC in treating resistant candidiasis. Further studies, including mammalian infection models and gene expression analyses, are warranted to fully evaluate the clinical potential of this combination.

## Data Availability

The original contributions presented in the study are included in the article/**Supplementary Material**. Further inquiries can be directed to the corresponding author.
