# Supplementary material for: Antifungal effects and potential mechanism of bortezomib in combination with fluconazole against Candida albicans
Source: Front Cell Infect Microbiol. 2026 Sep 17;16:1856948. doi: 10.3389/fcimb.2026.1856948 (PMC13627029; doi:10.3389/fcimb.2026.1856948)

**Figure S1** Anti-biofilm activity determined by CFU enumeration.

Biofilms of *C. albicans* CA10 ($1 \times 10^6$ CFU/mL) were treated with FLC (0.25 μg/mL), BTZ (8 μg/mL), or their combinations (0.25+8) for 4, 8, 12, and 24 h. Viable cells were quantified by CFU counting. Data are presented as mean ± SD from three independent experiments. *$P < 0.05$, ** $P < 0.01$, (two-way repeated-measures ANOVA with Dunnett's post hoc test).

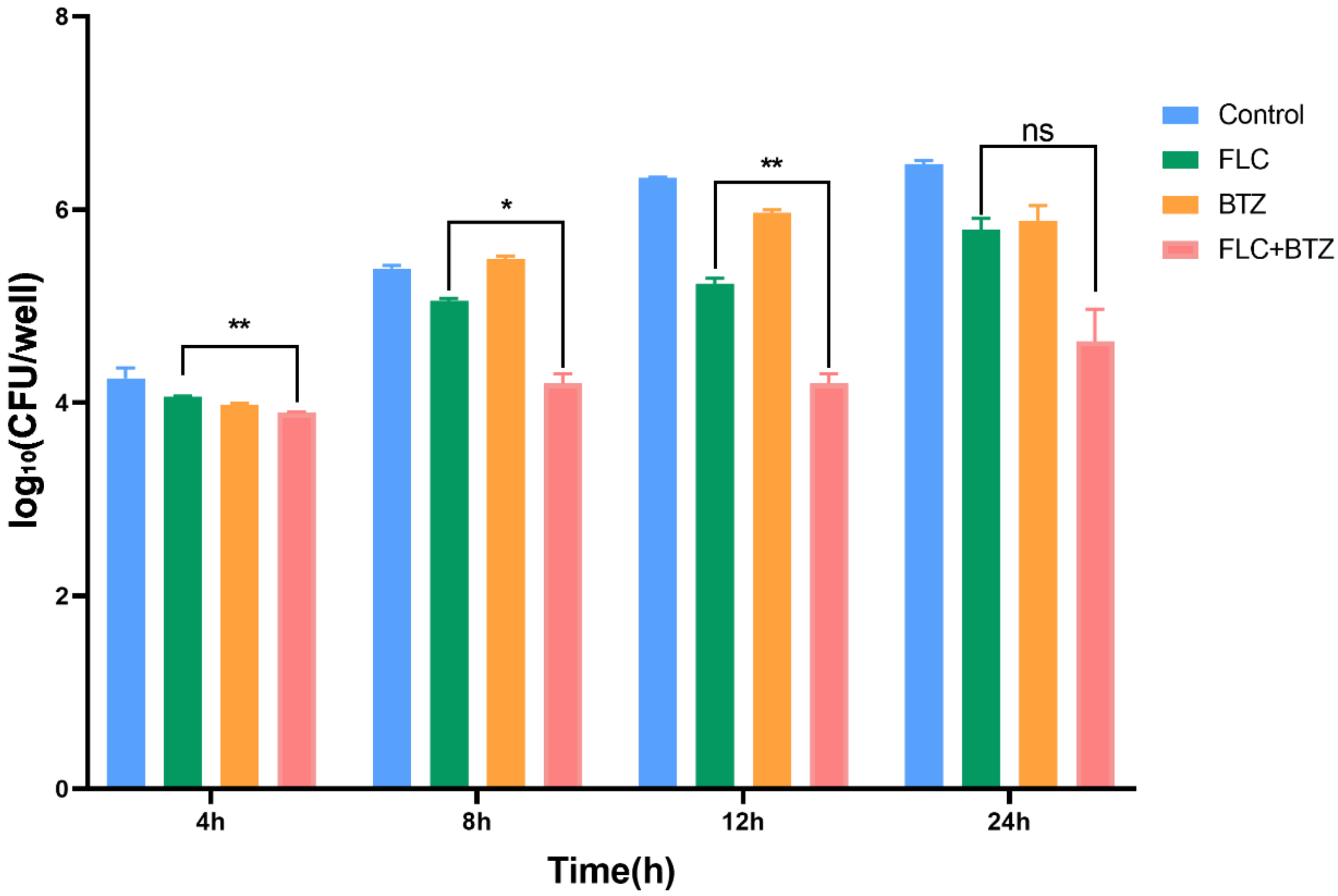

Supplement: Supplementary file 1 [file DataSheet1.pdf]
