# Supplementary material for: Antifungal effects and potential mechanism of bortezomib in combination with fluconazole against Candida albicans
Source: Front Cell Infect Microbiol. 2026 Sep 17;16:1856948. doi: 10.3389/fcimb.2026.1856948 (PMC13627029; doi:10.3389/fcimb.2026.1856948)

Figure S2. Quantification of hyphal-forming cells by manual counting in CA10 and CA16. Data are presented as mean ± SD from three independent experiments. *$P <$ 0.05, *** $P < 0.001$ vs. FLC group (one-way ANOVA with Sidak's post hoc test).

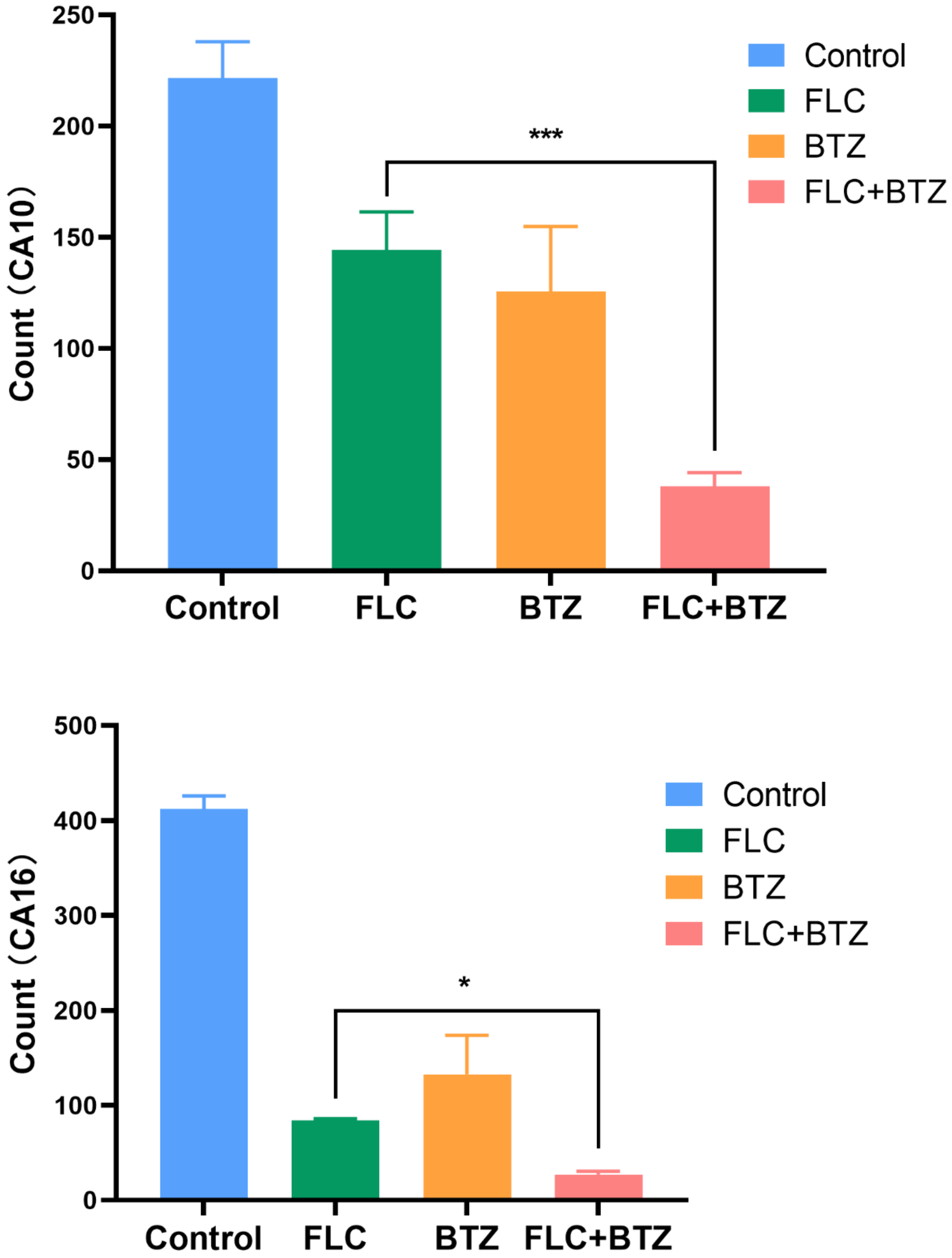

Supplement: Supplementary file 2 [file DataSheet2.pdf]
